# Spatial clustering of cholera cases in the Kathmandu Valley: implications for a ring vaccination strategy

**DOI:** 10.1093/inthealth/ihaa042

**Published:** 2020-08-06

**Authors:** Mellisa Roskosky, Mohammad Ali, Shyam Raj Upreti, David Sack

**Affiliations:** Department of International Health, Johns Hopkins Bloomberg School of Public Health, Baltimore, 615 N Wolfe Street, MD-21205, USA; Department of International Health, Johns Hopkins Bloomberg School of Public Health, Baltimore, 615 N Wolfe Street, MD-21205, USA; Group for Technical Assistance, Sanepa-3, Lalitpur, Nepal; Department of International Health, Johns Hopkins Bloomberg School of Public Health, Baltimore, 615 N Wolfe Street, MD-21205, USA

**Keywords:** cholera, Nepal, oral cholera vaccine, ring vaccination

## Abstract

**Background:**

In mid-2016, a cholera outbreak occurred in Kathmandu Valley, Nepal. This retrospective study aims to determine if a reactive, ring vaccination strategy would have been useful in preventing cholera transmission during that outbreak.

**Methods:**

Data on cholera cases were collected as part of hospital-based surveillance in the Kathmandu Valley in 2016. Global Positioning System (GPS) coordinates were obtained during household visits. Geographic clusters of cases were visually determined and tested statistically for clustering. Cluster size was determined based on the distribution of cases around the index case.

**Results:**

GPS coordinates for 69 cases were analysed. Six geographic clusters were identified, all of which showed significant clustering of cases. Approximately 85% of cases within a cluster occurred more than 7 d after the index case. The median ring size was 1 km, with a population of 14 000 people.

**Conclusions:**

Cholera cases were clustered in space and the majority of cases occurred over 1 week after the initial cases in the cluster, allowing for an opportunity to prevent transmission through the use of the vaccine soon after the initial case was identified. A ring vaccination strategy may be especially useful for large urban areas with recurrent seasonal outbreaks but where the specific locations for such outbreaks are not predictable.

## Introduction

Cholera remains a significant public health problem in several countries around South Asia.^1^ Six countries in the region are considered endemic for cholera, including Nepal, with an estimated 485 million people at risk. Of the 818 514 cases and 24 509 deaths estimated to occur annually in the region due to cholera, Nepal accounts for an estimated 30 379 and 911, respectively. Due to a lack of surveillance data, these estimates by Ali et al.^1^ are modelled based on the proportion of people in Nepal without access to improved water (11%) and sanitation (69%) facilities. Cases have been reported sporadically in multiple districts around the country and annually in the Kathmandu Valley over the last 5 y.^2^

Cholera transmission is environmentally driven by contaminated water bodies and seasonal monsoon rains and is subject to secondary spread through faecal–oral transmission.^3^ While the primary transmission is responsible for endemicity, it is this person-to-person spread that determines the magnitude of the outbreak.^4^ Plentiful data exist in support of cholera's spatial and temporal associations.^5–10^ The closer a person lives to a cholera case, the higher the risk of contracting the disease from that person. Thus cholera cases tend to be spatially clustered. This observation has led to recent research into the potential for controlling outbreaks via a ring vaccination strategy with a single dose of oral cholera vaccine (OCV). OCV has been shown to be effective in the prevention and control of cholera outbreaks in endemic settings.^11^ Two doses of OCV provides >80% protection during the first 6 months after vaccination. While a single-dose strategy lowers the effectiveness, it is more feasible in outbreak situations and has still been shown to provide significant short-term protection and potentially save more lives with fewer resources than the two-dose regimen.^12^ The evidence suggests that vaccinating a buffer of individuals around an index case, when implemented immediately upon case confirmation, could ultimately halt secondary transmission and prevent the disease from spreading to new areas.^6,12^

Nepal is known to be endemic for cholera, but limited surveillance data makes narrowing the target population through hotspot identification problematic. Despite these limitations, the endemic nature within Nepal and the explosive, epidemic potential of the disease given the plethora of risk factors existent in the Kathmandu Valley makes cholera a public health priority.

An outbreak of cholera occurred in the Kathmandu Valley during the summer monsoon months of 2016. In order to determine whether a reactive, ring vaccination would be an appropriate targeting strategy during an outbreak in Nepal, we aimed to explore the transmission patterns of cholera in space and time during the outbreak, with a particular focus on the spatial clustering of cases.

## Materials and methods

### Study area and population

**Figure 1.**
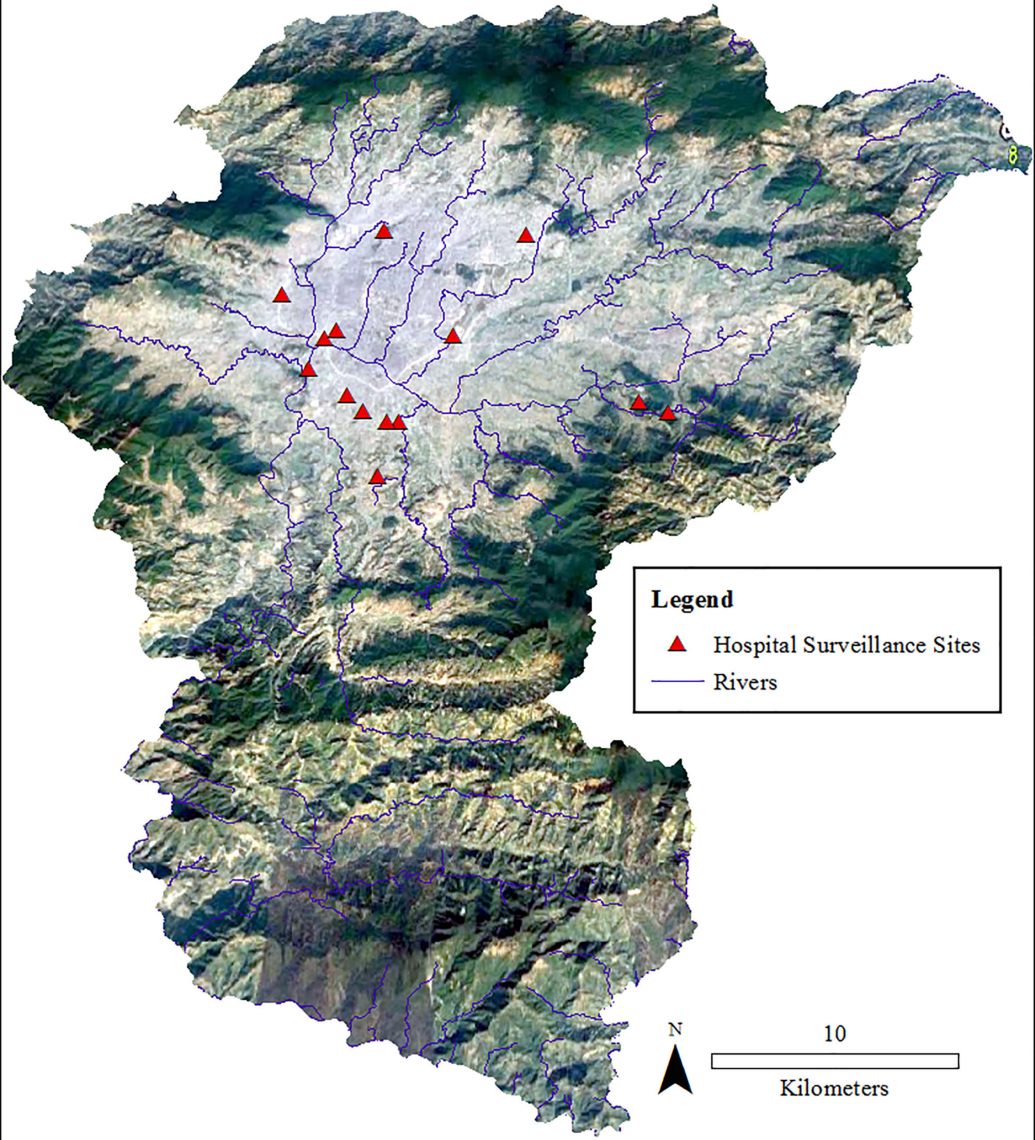
Study area, sentinel site hospitals and distribution of cases during the 2016 cholera outbreak.

The data were collected in Nepal's Kathmandu Valley, comprising three districts: Kathmandu, Lalitpur and Bhaktapur (Fig 1). It is the most developed area of Nepal and is surrounded by four mountain ranges. Outbreaks of cholera in Kathmandu Valley have historically occurred during the summer monsoon rains from June to August.^13–17^ As per the 2011 census, there were 119 wards in these three districts with a population of about 2.8 million.^18^

### Cholera data

Data on cholera cases were collected as part of hospital-based surveillance for cholera in the Kathmandu Valley from 30 June to 30 November 2016. A line listing, including hospital admission date, was maintained for all suspected cholera cases from the 14 chosen hospitals. Faecal specimens from the suspected cases were sent from the hospitals to the National Public Health Laboratory of the Ministry of Health (MOH) Nepal, which then notified the Ministry's Epidemiology and Disease Control Division (EDCD) of any culture-confirmed cases. A rapid response team then attempted to obtain permission for a visit to the home of the index case to collect additional information on the case.

### Geographic data

The geographic data of administrative units (district and ward boundaries) and rivers were obtained from the MOH. As part of the household investigation, Global Positioning System (GPS) locations of the case households were obtained using a mobile application (KoBo Toolbox, Cambridge, MA, USA) using a World Geodetic System (WGS) 1984 projection. The GPS surveyor was trained to utilize a standard length of time (10 min) to take the GPS readings. The time frame was chosen to ensure sufficient time for the GPS receiver to obtain required satellite signals to increase the positional accuracy of each reading. Receivers were held static and barrier-free while getting the readings. The GPS readings were then plotted on the ward map and on Google Earth imagery to ensure the accuracy of the data. In addition to case household locations, the locations of the health centres performing surveillance in the study area were collected using a similar technique.

To view and display the landscape of the study area, satellite imagery data of the study area was extracted from Google Earth Pro, which was then georeferenced in the same WGS 1984 projection so that the imagery could be superimposed onto a digital map of the study area.

### Spatial analysis

The geographic clusters of the cholera outbreak were defined within the study area using visual analysis. The outbreak's index case (the first case inside the study area), along with the index case in each cluster (first case within the cluster) were identified by hospital admission date. Linear distances (meters) and times (days) of the secondary cases were calculated in relation to the location and date of presentation of the index case in a cluster. Before calculating linear distance, the geographic data were transformed from the WGS 1984 projection to a Universal Transverse Mercator Zone-45 North projection. The distance and time of the index case of each cluster from that of the outbreak index case was also calculated. Cases were plotted based on distance and time in order to postulate transmission dynamics within a cluster.

Spatial clustering of the cases was assessed using Ripley's K function for the cholera case household locations. The K function estimates the expected number of additional cases within a range of distances of other cases. To account for the effects of complete spatial randomness, the L function, which is a variance-stabilized transformation of the K function, was also plotted. The estimates were plotted as a function of distance along with a Monte Carlo assessment of complete spatial randomness to account for uncertainty. Both K and L function analyses were performed using ‘spatstat’ implemented in the R statistical software (R Foundation, Vienna, Austria) and mapping was done using ArcGIS 10.5.1 (Esri, Redlands, CA, USA).

The maximum distance from the index case to a subsequent case was recorded for each cluster. This was done for cases occurring within 14 d of the index, reasoning that further spread of transmission would have been stopped occurring within 14 d of the index case. Circular rings around each cholera index case were drawn using the median distance of the clusters. The underlying population within each ring was estimated based on the area of the ring overlapped with the area of the ward. Note that the ward is the lowest administrative unit of the Ministry of Federal Affairs and Local Development in Nepal. The ward population was based on the Nepal's 2011 national census. The following equation was used to calculate the population for each ring:

$$\begin{equation*}
{p_j} = \mathop \sum \limits_{i = 1}^n {w_i} \times \frac{{{r_j}}}{{{a_i}}},
\end{equation*}$$

where *p_j_* is the population in ring *j, w_i_* is the population in ward *i, r_j_* is the ring *j* area overlapped with the ward *i* area and *a_i_* is the area of ward *i*. The calculations were performed using R statistical software (R Foundation).

## Results

**Figure 2.**
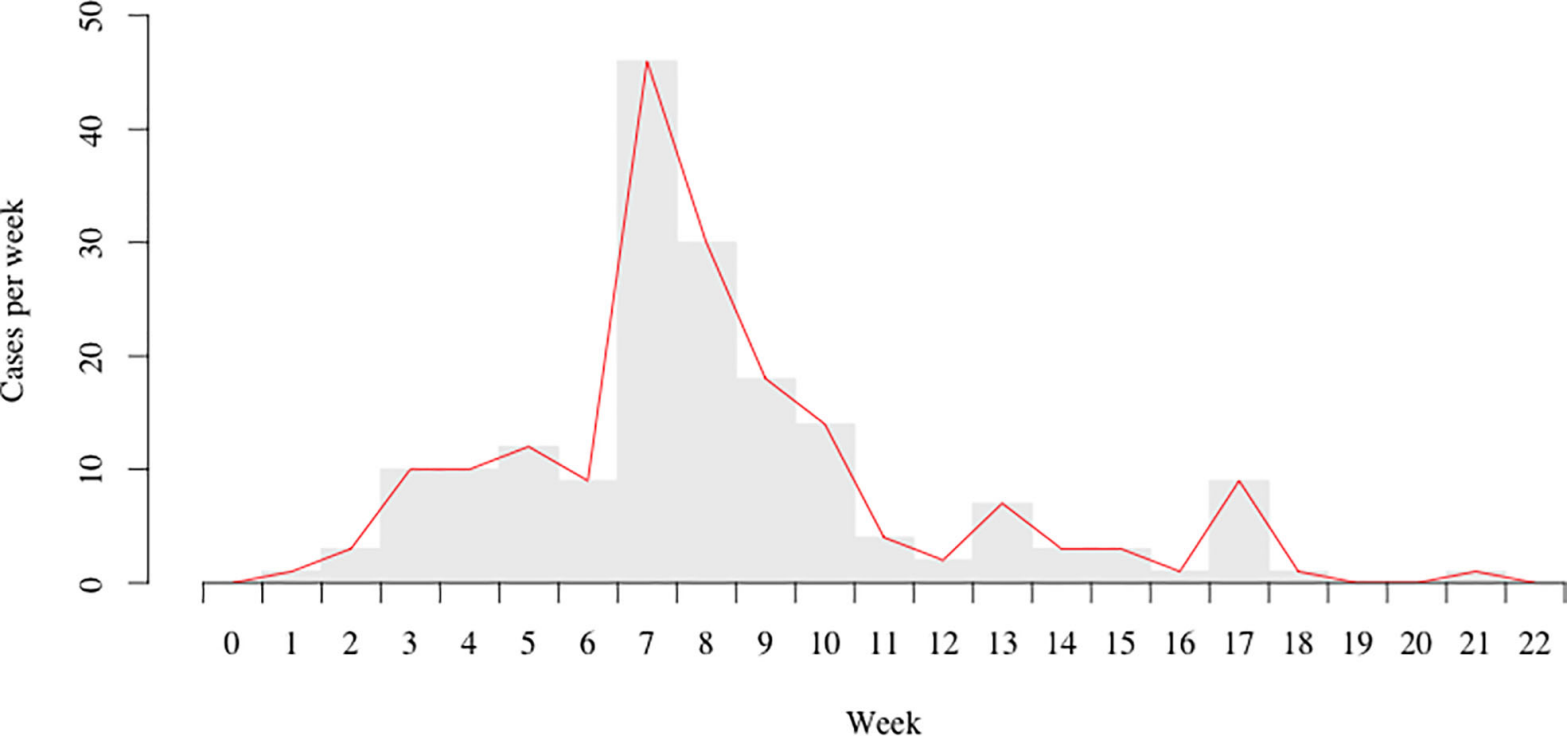
Epidemic curve of the cholera outbreak in Kathmandu Valley, 2016. Cases are shown per week. Curve includes all 193 confirmed cases during the outbreak.

**Figure 3.**
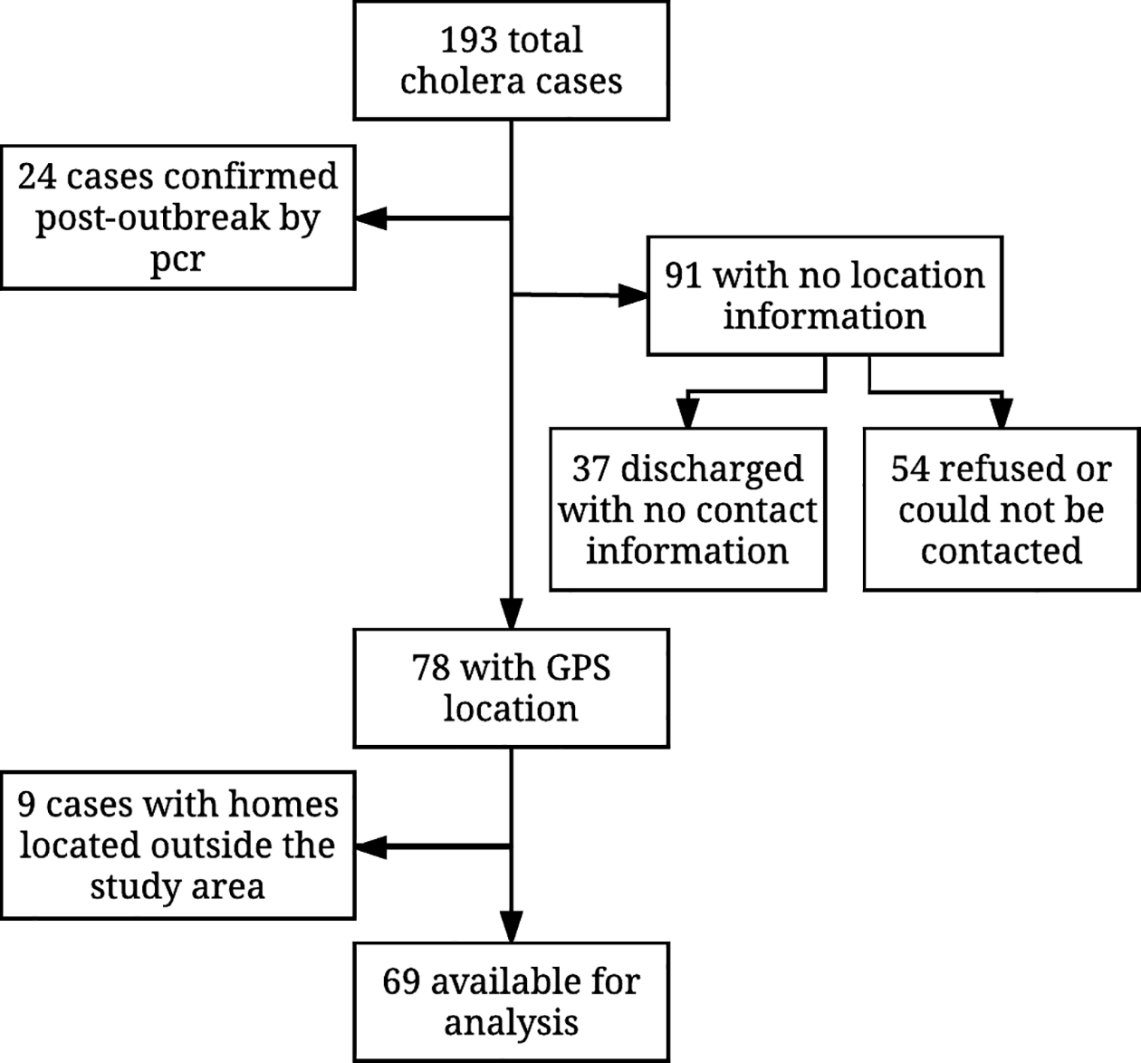
Data flow chart. A total of 69 cases were included in the spatial analysis.

During the outbreak, a total of 193 cases of *Vibrio cholerae* O1 were reported to the MOH in the Kathmandu Valley through the hospital-based surveillance system. The epidemic curve shows the peak period of the outbreak was 7–10 weeks from the date of the initial case of the outbreak (Fig. 2). Of 193 cases, the GPS coordinates of the case households were available for 78 households, of which 9 locations were outside the study area, leaving 69 cases for the spatial analysis (Fig. 3).

**Figure 4.**
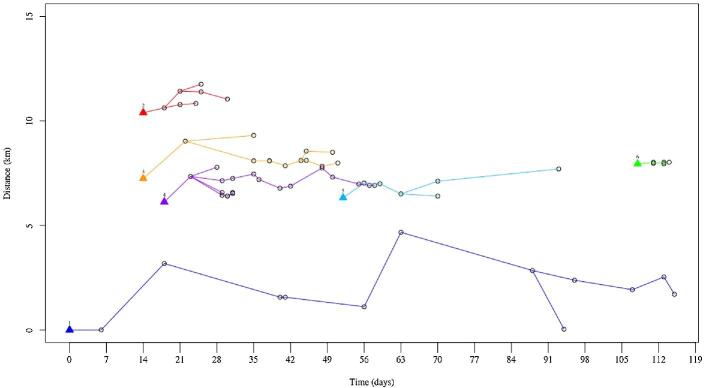
Diffusion of cholera in space and time in the different geographic clusters in Kathmandu Valley, 2016. Triangles indicate a cluster index, along with the cluster number. Each point represents a case, with lines indicating potential transmission over time.

**Figure 5.**
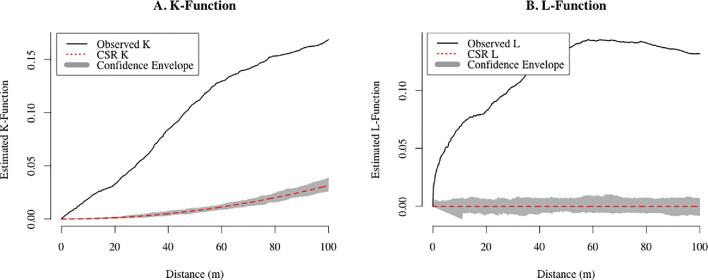
K and L functions for global spatial clustering (based on 69 cases). Black lines indicate the degree of clustering that was observed in the data. Red lines indicate what would be expected if cases were completely spatially random. The grey envelope represents the 95% confidence interval.

The distribution of those 69 cases was plotted on a map that clearly shows spatial patterning of the cases (Fig. 1). Using visual analysis of the spatial distribution of the cases and transmission patterns in space and time (Fig. 4), six geographic clusters were defined. Global and local (within each of the clusters) clustering of the cases was supported by the Ripley's K and L functions (Fig. 5 for global clustering and Fig. S1 for local clustering of the cases). In the local cluster analysis, two cases were excluded for which the locations were deemed outliers, as they did not fall within any of the six clusters.

**Figure 6.**
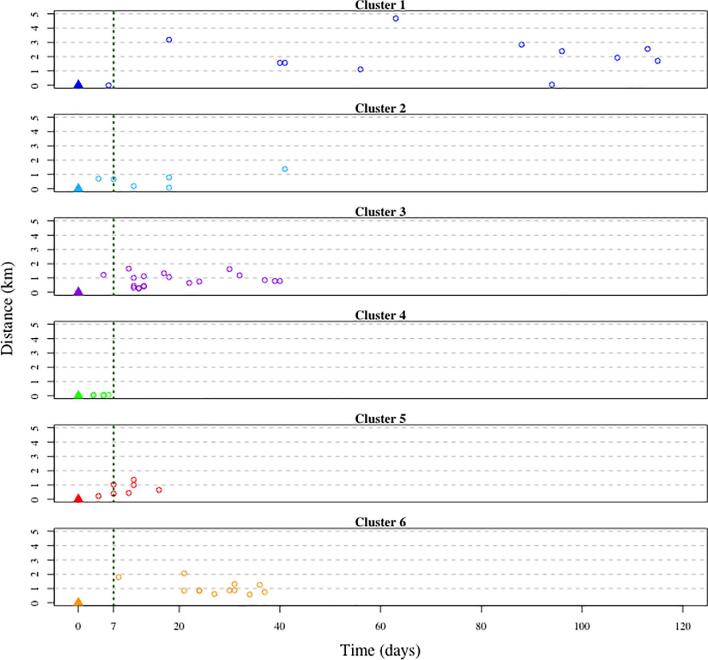
Spatial and temporal dynamics of cholera cases by cluster. Distance and time are relative to the cluster index case.

An average of 11 cases were observed in a cluster, with a median cluster duration of 38 d (Table 1). The median of the average distance of the subsequent cases in each cluster from the initial case was 0.79 km (Table 1). A total of 85% (52/61) of cases within a cluster occurred more than 7 d after an index case reported to the hospital (Fig 6) and 44% (27/61) were within 14 d of the presentation. The median of the maximum distance of a subsequent case within 14 d of the initial case in the cluster was 1 km (Table 2). Population sizes ranged from 9917 people in cluster 2 to >65 000 in cluster 3, with a median population of 14 000 within 1 km of the index case of a cluster (Table 2).

**Table 1.** Spatiotemporal characteristics of cholera case clusters

		Time (days)		Distance (km)	
Cluster ID	Cases, n	Outbreak onset to cluster onset	Cluster duration	Primary index to cluster index	Cluster index to subsequent cases (average)
1	13	0	115	0.00	1.96
2	8	14	16	10.39	0.73
3	13	14	37	7.24	1.05
4	20	18	40	6.12	0.86
5	7	52	41	6.32	0.64
6	6	108	6	7.95	0.05
Median	10.50	18.00	38.50	7.24	0.79

**Table 2.** Maximum distance within 14 d of the index case and estimated population by cluster

Cluster	Maximum distance (km)	Estimated population within 1 km
1	0.0003	53 341
2	0.703	9917
3	1.66	65 134
4	0.076	16 117
5	1.363	10 686
6	1.79	11 891
Median	1.033	14 004

### Discussion

In this outbreak, the majority of subsequent cases were infected 1 week after the initial case in each cluster. This suggests that subsequent cases could have been prevented in each of these clusters had a vaccination program been initiated soon after the initial case in that area was identified. While the second reported case in the outbreak occurred in the same area within 1-week's time and could have been exposed from a local contaminated water source, the three subsequent cases occurred 2 weeks after the initial case presentation and were far (≥5 km) from the initial case. This pattern, along with the significant spatial clustering observed in the data, indicates that the outbreak likely followed the typical pattern of primary infections from a contaminated water source, occurring almost simultaneously in different areas of the valley and leading to secondary person-to-person propagation in distinct clusters via faecal–oral transmission.^3^ So while contaminated water sources from monsoon rains could be deemed responsible for the cluster index cases, the bulk of the outbreak is likely determined by poor hygiene and sanitation conditions.^3,24^ These results have practical importance in terms of the types of interventions that should be used to control cholera in Nepal, as well as how they should be targeted. Using vaccines in this scenario has the potential to break the secondary transmission chain and limit the spread of the outbreak.

Within a cluster, the majority of cases were detected >7 d after the cluster index case. However, evidence from another cholera-endemic area suggests that the first week after any cholera case is a high-risk period for neighbours of that case.^5^ There are no data on the difference in transmission mechanisms between endemic and outbreak settings, however, this high-risk period highlights the importance of delivering additional interventions within this vulnerable period. There are strong calls for the use of water, sanitation, and hygiene (WASH) interventions coupled with OCV in these settings. However, there is little direct evidence on how to integrate effective WASH programs into an urgent vaccination program such as ring vaccination. A recent study in Dhaka, Bangladesh found that the addition of a large-scale WASH program to a vaccination campaign did not reduce the number of diarrhoea-related hospitalizations^25^, however, a WASH intervention focused on the family of an index case was effective in reducing symptomatic *V. cholerae* infections.^26^ Similarly, a recent meta-analysis demonstrated the protective effect of prophylactic antibiotics in preventing cholera among exposed household contacts, while other research warns of the risks of resistance over the course of an outbreak.^27–29^ More robust research is needed to design impactful interventions for this high-risk period and to focus these interventions on the high-risk neighbourhoods near the index cases. Given that in this study the majority of the cases occurred 1 week after the initial case, vaccinating people surrounding the case could effectively reduce the spread of cholera in this instance.

Cases occurring within 14 d of the initial case in a cluster also occurred within 1 km of that index case. This suggests that ring vaccination 1 km around the index case would be an effective strategy in stopping transmission of the disease, assuming immediate action is taken by the MOH and that OCV is likely to provide immunity within 7 d after immunization.^22,23^ Such a vaccination strategy would also be highly cost effective to limit cholera transmission in densely populated urban areas by reducing the number of doses usually used to combat an outbreak.^6^ The Kathmandu Valley has a population of >2.5 million, and while the amount of available vaccine has increased dramatically over the last few years, demand continues to far exceed supply. Even in the event that the vaccine supply was limitless, vaccinating the entire population would remain logistically and financially challenging.

The rings with the highest populations in this study overlapped with wards of the Kathmandu and Lalitpur metropolitan cities, which have a combined population of 1.2 million. Based on the estimated underlying population in the ring, strategies may need to be designed differently for the metropolitan areas, where a high population density may lead to high risk and would require a large amount of vaccine to be available. The average household size for urban areas in the central development region, of which Kathmandu Valley is a part, is approximately 4.24.^18^ Thus the MOH could anticipate visiting approximately 3300 households (14 000 persons) per ring in clusters to administer approximately 14 000 doses of vaccine with a single-dose strategy.

Although the outbreak continued for approximately 4 months and exhibited atypical seasonality by continuing well after the conclusion of monsoon season, the peak was of short duration (4 weeks), as has been found in many other situations in Nepal.^19–21^ The risk for hospitalization due to cholera was highest during weeks 7–10 after the onset of the outbreak. These observations should inform preparation activities and resource allocation over the course of future outbreaks, such as ensuring additional personnel are available for investigations and interventions in the community during the peak period or that stockpiles of essential supplies are large enough to see response teams through an entire season.

While this analysis was performed on a detailed dataset, the data are limited to a single outbreak, making it difficult to generalize these results to what can be expected in the coming years. It will be important to continue to monitor the transmission dynamics during future outbreaks to better refine control strategies. The analysis is also limited by the nature of the surveillance data being hospital-based, as it is likely that cases were missed within the community. Kathmandu Valley has many health facilities, but few with the capability for bacterial culture, which was a criteria for case confirmation. While sentinel site hospitals were chosen in an attempt to cover the entire Kathmandu Valley population, travel to these hospitals would be an impractical distance for many households on the outskirts of the valley. Even for those making it to the hospital sites, not all cases consented to household follow-up. This limited the number of cases with complete spatial data and therefore does not show the full extent of the outbreak or size of the clusters. The median number of cases per cluster was approximately 10. It is difficult to draw conclusions on transmission dynamics within the smallest clusters without spatial data on the additional cases. Finally, case clusters were defined visually, not adjusting for the underlying population. It is therefore possible that the spatial pattern of cases seen here is biased by the spatial pattern of the population.

In this study, the spatiotemporal dynamics of a single cholera outbreak in the Kathmandu Valley were investigated. Cholera cases during the outbreak clustered in time and space, a pattern that presents an opportunity to prevent further spread via ring vaccination. This analysis is a valuable contribution to the evidence in support of the design of a cholera control strategy; however, the success of such an approach requires vaccine to be readily available and immediately deployed upon case confirmation. The effort required for advanced preparation would be repaid by the prevention of both immediate infections and subsequent transmission of cholera to neighbouring individuals and areas. It is likely that this strategy would require even fewer doses in a rural area outside the densely populated Kathmandu Valley, although further research is needed to support this hypothesis. Understanding that the spread of cholera in the Kathmandu Valley is predominately driven by secondary transmission highlights the potential for substantial spread of the disease in a disaster situation and the importance of improving water and sanitation conditions throughout the country. As annual surveillance data continues to be collected on cholera in the Kathmandu Valley, it will not only allow for validation of these findings, but more in-depth analysis can be performed to better understand the ‘typical’ outbreak in terms of size, household risk factors, seasonality and identification of hotspots. This type of detailed longitudinal data will allow health officials to further hone the country's preparedness activities.
